# CNVIntegrate: the first multi-ethnic database for identifying copy number variations associated with cancer

**DOI:** 10.1093/database/baab044

**Published:** 2021-07-14

**Authors:** Amrita Chattopadhyay, Zi Han Teoh, Chi-Yun Wu, Jyh-Ming Jimmy Juang, Liang-Chuan Lai, Mong-Hsun Tsai, Chia-Hsin Wu, Tzu-Pin Lu, Eric Y Chuang

**Affiliations:** Bioinformatics and Biostatistics Core, Center of Genomic and Precision Medicine, National Taiwan University, Taipei 10055, Taiwan; Graduate Institute of Biomedical Electronics and Bioinformatics, National Taiwan University, Taipei 10617, Taiwan; Graduate Institute of Biomedical Electronics and Bioinformatics, National Taiwan University, Taipei 10617, Taiwan; Cardiovascular Center and Division of Cardiology, Department of Internal Medicine, National Taiwan University Hospital, Taipei 10008, Taiwan; College of Medicine, National Taiwan University, Taipei 10051, Taiwan; Bioinformatics and Biostatistics Core, Center of Genomic and Precision Medicine, National Taiwan University, Taipei 10055, Taiwan; Graduate Institute of Physiology, National Taiwan University, Taipei 10051, Taiwan; Bioinformatics and Biostatistics Core, Center of Genomic and Precision Medicine, National Taiwan University, Taipei 10055, Taiwan; Institute of Biotechnology, National Taiwan University, Taipei 10672, Taiwan; Center for Biotechnology, National Taiwan University, Taipei 10672, Taiwan; Graduate Institute of Biomedical Electronics and Bioinformatics, National Taiwan University, Taipei 10617, Taiwan; Bioinformatics and Biostatistics Core, Center of Genomic and Precision Medicine, National Taiwan University, Taipei 10055, Taiwan; Department of Public Health, Institute of Epidemiology and Preventive Medicine, National Taiwan University, Taipei 10055, Taiwan; Bioinformatics and Biostatistics Core, Center of Genomic and Precision Medicine, National Taiwan University, Taipei 10055, Taiwan; Graduate Institute of Biomedical Electronics and Bioinformatics, National Taiwan University, Taipei 10617, Taiwan; Master Program for Biomedical Engineering, China Medical University, Taichung 40402, Taiwan

## Abstract

Human copy number variations (CNVs) and copy number alterations (CNAs) are DNA segments (>1000 base pairs) of duplications or deletions with respect to the reference genome, potentially causing genomic imbalance leading to diseases such as cancer. CNVs further cause genetic diversity in healthy populations and are predominant drivers of gene/genome evolution. Initiatives have been taken by the research community to establish large-scale databases to comprehensively characterize CNVs in humans. Exome Aggregation Consortium (ExAC) is one such endeavor that catalogs CNVs, of nearly 60 000 healthy individuals across five demographic clusters. Furthermore, large projects such as the Catalogue of Somatic Mutations in Cancer (COSMIC) and the Cancer Cell Line Encyclopedia (CCLE) combine CNA data from cancer-affected individuals and large panels of human cancer cell lines, respectively. However, we lack a structured and comprehensive CNV/CNA resource including both healthy individuals and cancer patients across large populations. CNVIntegrate is the first web-based system that hosts CNV and CNA data from both healthy populations and cancer patients, respectively, and concomitantly provides statistical comparisons between copy number frequencies of multiple ethnic populations. It further includes, for the first time, well-cataloged CNV and CNA data from Taiwanese healthy individuals and Taiwan Breast Cancer data, respectively, along with imported resources from ExAC, COSMIC and CCLE. CNVIntegrate offers a CNV/CNA-data hub for structured information retrieval for clinicians and scientists towards important drug discoveries and precision treatments.

**Database URL**: http://cnvintegrate.cgm.ntu.edu.tw/

## Introduction

Human copy number variations (CNVs) or copy number alterations (CNAs) are DNA segments greater than 1000 base pairs (bp) that are duplicated or deleted with respect to the reference genome (1). CNVs are variations that are inherited through germline cells, whereas CNAs are acquired somatic changes that lead to gain or loss of copies of DNA segments. These duplications and deletions can potentially alter gene expression levels (2) and cause diseases such as cancer, viral infection disorders, neuropsychiatric diseases and obesity (3–6) through gene dosage, gene disruption, gene fusion or positional effects (7). CNVs are a source of genetic diversity in healthy populations (8–10) and are predominant drivers of gene and genome evolution. With the advent of high-throughput techniques, the International HapMap Consortium (11), the 1000 Genomes Project (12) and other large-scale whole-genome studies have identified a large number of CNV segments that are the cause of 4.8–9.5% of the variability in the human genome (13, 14). To distinguish CNVs representing benign polymorphic variants from disease-causing CNVs, one potential strategy is to compare genomes of healthy and diseased individuals. To this end, a convincing reference panel of CNVs that contribute to human genetic diversity, may or may not convey phenotypes, needs to be constructed.

Early studies demonstrated that CNV landscapes differ significantly among ethnicities (15–17). In the past, several CNV reference panels have been built, such as the ones for Caucasian and African American control samples (18, 19). Several large-scale datasets, such as the 1000 Genomes Project (12), the Jackson Heart Study (20) and the NHLBI’s GO Exome Sequencing Project (21), have been constructed as efforts to study the CNV landscape of healthy individuals from different countries, serving as global reference genomes (6, 22). Whole exome sequencing (WES) data from 17 such international projects were aggregated into the Exome Aggregation Consortium (ExAC), which catalogs DNA sequence variants, including CNVs, of nearly 60 000 healthy individuals across five demographic clusters [European, African, South Asian, East Asian and admixed American (Latino)] (23). However, one caveat of such studies is the small sample sizes, representing each sub-population (24, 25). Availability of a CNV panel with a larger representation of a healthy population, such as Taiwanese/Chinese, would facilitate researchers with a baseline to identify disease-associated CNVs from benign ones, for the East Asian subpopulation.

CNAs associated with cancer susceptibility have been broadly characterized over the years and have successfully revealed distinct CNA profiles in the genomes of cancer cohorts (26–31). For instance, Lu *et al.* reported frequent copy number (CN) altered regions in at least 30% of non-smoking female lung adenocarcinoma patients through a genome-wide CNV analysis (29), and Li *et al.* utilized 266 CN probes to show that CNA landscapes vary between lung cancer subtypes, thus highlighting the potential of CNAs as biomarkers (31). To further explore the role that CNAs play, in the etiology of human cancers, large projects were launched. One of the most comprehensive resources is the Catalogue of Somatic Mutations in Cancer (COSMIC) (32), which combines CNA data from the International Cancer Genome Consortium (33) and The Cancer Genome Atlas (https://www.cancer.gov/about-nci/organization/ccg/research/structural-genomics/tcga) (34). Another collaborative project for CNA exploration, the Cancer Cell Line Encyclopedia (CCLE) (35), aims to conduct detailed genetic and pharmacological characterizations using a large panel of human cancer cell lines. However, we still lack a structured and comprehensive CNV/CNA resource from healthy individuals and cancer patients, respectively, across large populations.

In this study, we, first, aimed to characterize CNV profiles in the general population, living in Taiwan and construct a reliable Taiwanese CNV reference panel (TWCNV) using ∼15 829 DNA samples from the Taiwan Biobank (36). This reference panel would be a potential resource against which to conduct comparison analyses of causal CNVs in disease-related studies of Taiwanese patients. Second, we included a CNA panel, Taiwanese Breast Cancer (TWBC), consisting of CNA profiles from 114 breast cancer patients in Taiwan. This CNA panel would allow users to evaluate whether a cancer-related variant is population dependent or not. Finally, we present CNVIntegrate, a user-friendly web-based system with an integrated, sorted and structured CNV/CNA database built from multiple sources: TWCNV, ExAC, TWBC, COSMIC and CCLE. The former two datasets consist of samples from healthy general populations, while the latter three datasets contain cancer-associated CNAs. CNVIntegrate provides statistically comparable metrics to predict the functional impact of a CNA by comparing the healthy person data with the cancer patient data. There are a few CNV databases, such as the Korean Variant Archive (37), the Pan-Asian single nucleotide polymorphism (SNP) Genotyping Database (38) and ThaiCNV (39) that host CNV information from healthy individuals from different countries and regions, but CNVIntegrate is the first to offer comprehensive support for identifying cancer-associated CNAs in a multiethnic population.

## Material and methods

### Database overview

**Figure 1.**
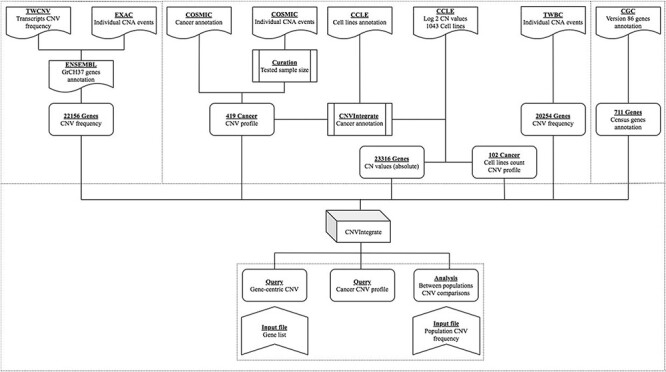
Overview of CNVIntegrate.

An overview of the system is illustrated in Figure 1. Table 1 provides a thorough comparison of features of CNVIntegrate to those of currently available CNV databases. To the best of our knowledge, this is the only web-based system that hosts CNV and CNA data from both healthy populations and cancer patients, respectively, and concomitantly provides functional scores by performing statistical comparisons of CNV frequencies among multiple populations. CNVIntegrate is an easily accessible data hub that supports the retrieval of structured CNV information by a single query or a file upload.

**Table 1.** Comparison of sample sizes, functions, and query results offered by existing databases

		Regional databases			Global databases		
	CNVIntegrate^a^	KOVA	PanSNPdb^d^	ThaiCNV^e^	ExAC	COSMIC^f^	CCLE^g^
Population	Taiwanese	Korean	Asian	Thai	NA	NA	NA
Multi-ethnic	+				+	+	+
Population sample size	15 943^b^	1055	1719	3017	NA	NA	NA
Sample size	1 105 891 ^c^	1055	1719	3017	59 898	^f^	^g^
Gene query	+	+			+	+	
Chromosomal query	+	+	+		+	+	
Batch query	+						
Downloadable data	+	+			+	+	+
Global reference	+				+		
Comparison analysis^h^	+						

a CNVIntegrate includes individuals from Taiwan (TWCNV), ExAC, TWBC, COSMIC and CCLE. TWCNV consists of CNV data from healthy individuals of Taiwanese cohort, ExAC consists of CNV data from healthy individuals from multiple cohorts, TWBC consists of CNV data from breast cancer patients in Taiwanese cohort and COSMIC and CCLE consists of CNV data of cancer-affected individuals from multiple cohorts.

b 15 829 healthy samples and 114 Breast cancer samples from Taiwanese cohort.

c CNVIntegrate includes healthy individuals and breast cancer patients from Taiwan cohort along with samples from ExAC, COSMIC and CCLE.

d PanSNPdb included individuals from China, India, Indonesia, Japan, Malaysia, Philippines, Singapore, South Korea, Taiwan and Thailand.

e ThaiCNV provides direct visualization of CNV data as tracks embedded on the UCSC Genome Browser. Individual CNA events detected among samples are shown without additional information such as CNV frequencies, gene annotation, etc.

f COSMIC hosts 1 179 545 CNVs collected by expert and exhaustive curation of over 26 000 publications.

g CCLE data included CNVs of 3316 genes across 1043 cell lines. CNV data from CCLE are not available online; data need to be downloaded and processed for further application.

h Comparison of CNV frequencies amongst different ethnic populations.

**Figure 2.**
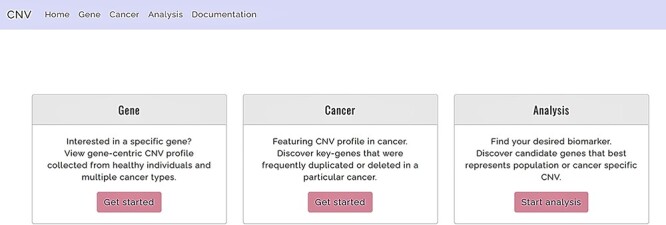
Functions offered by CNVIntegrate—gene, cancer and analysis (i) Gene: gene-query function. (ii) Cancer: cancer profile function. (iii) Analysis: analysis function.

The system presents the users with three major functions (Figure 2), namely, (i) the gene-query function, (ii) CNV profile function for specified cancer and (iii) the analysis function. The gene-query function provides users with CNV information for the queried gene/chromosome/region of interest. Using the CNV profile function, the users can further choose from a list of cancer types to obtain CNA profiles for a specified cancer. The analysis function offers the users a comparison analysis for CNV frequency among healthy and affected populations across different ethnicities. It potentially isolates cancer-associated genes with a CNV prevalence that deviates from the normal baseline population and also provides an accurate clinical interpretation of the CNV, through multiple, between-population comparisons, using CNV/CNA information from both healthy individuals and cancer patients. The web site supports display on mobile devices as well as mainstream internet browsers, such as Google Chrome, Apple Safari and Internet Explorer. The database was developed with Django 2.1 and runs on Python programming language version 3.4 and MySQL. Users are offered unlimited search options, along with easily retrievable search results.

### Database content

CNVIntegrate creates a comprehensive CNV and CNA landscape by compiling data from healthy individuals and cancer patients, respectively. CNV data for healthy cohorts are imported from the TWCNV (created and characterized in this study, see below) and ExAC databases (23). Cancer-specific CNA datasets such as TWBC (details, see below), COSMIC (32) and CCLE (35) are imported into the dataset. All included datasets in CNVIntegrate are converted to the human genome assembly version 37(GRCh37) for consistency. Furthermore, genes and their respective annotation data are imported from the Cancer Gene Census (CGC) (https://cancer.sanger.ac.uk/census) (40).

#### TWCNV reference panel

##### Datasets used in TWCNV baseline construction and comparison

To characterize a CNV reference panel specific for the Taiwanese sub-population, SNP array data from peripheral blood samples of 15 829 volunteers without cancer living in Taiwan were included in this study. The SNPs were assayed using an Axiom Genome-Wide TWB Array Plate (TWB array), one of the Affymetrix Axiom Genotyping systems designed for the Taiwan Biobank. The TWB array contains 653 291 SNP probes for SNPs specific to people of Taiwan and 525 652 SNP probes from the Axiom Genome-Wide CHB Array for SNPs related to diseases and drug metabolism that have been reported in previous studies (41). TWCNV contains 476 247 CN alterations identified on autosomal chromosomes, encompassing more than 20 000 genes. To confirm the credibility of our TWCNV results, three public SNP array datasets were used for comparison. The first one was from the International HapMap Project (42) including 267 blood samples that were assayed on the Mapping 500K array platform and contains a similar number of SNP probes to that of the TWB array. The 267 samples comprise 30 Caucasian trios (two parents and one child), 30 African trios and 87 unrelated Asian individuals. Two other datasets, downloaded from the Gene Expression Omnibus, of which GSE30481 contains blood samples of 155 general Chinese individuals (87 Han Chinese, 44 Tibetans, 9 Dongs, 8 Yaos, 6 Zhuangs, 8 Lis and 4 Uyghurs) (43) and GSE23291 consists of blood samples from 490 Ashkenazi Jews, were used as controls in this study (44).

##### Genotyping, quality control (QC) and quality assessment

Genotype calling was performed using Affymetrix Power Tools version 1.18.0 (https://www.thermofisher.com/tw/zt/home/life-science/microarray-analysis/microarray-analysis-partners-programs/affymetrix-developers-network/affymetrix-power-tools.html). A two-step sample QC was conducted, where, first, a statistic (Dish-QC) was created to measure the signal across non-polymorphic loci in the samples. Samples that achieved a (Affymetrix suggested) default Dish-QC >82% were then checked for the sample call rate (CR). Samples that exceeded a CR default cut-off value of 97% were identified and retained for further downstream analysis. The quality of the genotyping results was then evaluated by conducting a comparison of the gender information predicted from the allosome genotyping results with that of the real gender information provided by Taiwan Biobank for all the samples that passed the QC steps.

##### CNV calling, CNV analysis and gene annotation

The Partek Genomics Suite 6.6 (https://www.partek.com/partek-genomics-suite/) was used for CNV detection. The results from the genotyping step were quantile normalized and compared to the reference (created by selecting all samples from our TWB data) using the Partek Genomics Suite. The Partek algorithm assumes that the median of the intensity values for each SNP, over all TWB samples, represents an average CN of 2. CN calls were made by comparing intensity values of each test sample to the pooled reference sample. CNV regions were called based upon a few predefined criteria: minimum count of consecutive genomic markers ≥ 35, *P*-value ≤ 0.001 and signal-to-noise ratio ≥ 0.3. Theoretically, a genomic segment was called a CNV when its average CN change was higher than 0.3 (amplified regions CN > 2.3; deleted region CN < 1.7) with a significant *P*-value using a *t*-test (29). Since the resolutions of the 500K arrays and SNP 6.0 arrays are ∼0.75 and four times greater, respectively, than that of the TWB array, the minimum consecutive genomic markers required to define a CNV region were adjusted to ≥25 and ≥100 for the two array platforms, with the other parameters unchanged.

The CNV regions across the samples were analyzed after the individual CNVs were detected. A Python script was utilized to analyze the CNV segments, by identifying a common region between any two breakpoints across all the samples. Consequently, the frequencies of any common region were calculated in the general Taiwanese population. The Reference Sequence Database (45) was then utilized to map these segments onto gene symbols using the ‘nearest features’ function embedded in the Partek Genomics Suite, for exploring their potential functions. The genes encompassed by CNVs were those with at least one overlapping nucleotide from each CNV segment. To further fine-map CNVs to the gene level, the highest duplication/deletion frequencies of all the segments located in a single gene were utilized. Hence, the baseline CNV frequency of genes serving as benchmarks was established. This study specifically focuses on the autosomes. All CNV analyses and downstream characterizations were done using the human genome version 19 (hg19) build.

#### ExAC dataset

ExAC is a worldwide project that aims to assist in the functional interpretation of variants by aggregating and harmonizing whole exome data generated by 17 sequencing projects including 1000 Genomes (12), the Jackson Heart Study (20), NHLBI’s GO Exome Sequencing Project (21) and others. The ExAC dataset catalogs DNA sequence variants including 126 771 rare CNVs from 59 898 healthy individuals spread across five demographic clusters, European, African, South Asian, East Asian and admixed American (Latino), thereby representing a worldwide control cohort for CNV reference analysis (23, 35). CNVIntegrate includes CNV data from ExAC version 0.3.1. In this study, the CNV regions from ExAC were mapped onto the official HUGO Gene Nomenclature Committee gene symbols and thereby global CNV frequency, as well as population-specific CNV frequencies, was calculated for each gene, to be included in the CNVIntegrate database.

#### TWBC dataset

TWBC consists of CNA profiles from 114 Taiwan breast cancer patients with primary breast cancer. Subjects recruited for this dataset include a subset of clinically diagnosed breast cancer patients who undergo surgical resection at four different hospitals in Taiwan (Lotung Poh-Ai Hospital, Cathay General Hospital, Kaohsiung Medical University Hospital, and Cheng Ching Hospital). Matched tumor and adjacent normal tissues were collected and sequenced using WES, and CNA analysis was performed by FACETS (46) and GISTIC2 (47).

#### COSMIC dataset

COSMIC is one of the most comprehensive resources for exploring mutations and structural variations in human cancer, hosting 1 179 545 CNAs curated over 26 000 publications where CN data (32) have been collected from the International Cancer Genome Consortium (33) and The Cancer Genome Atlas (34). Sample level information from COSMIC, including CNV gain or loss events, site of sample origin, sample histology and cancer classification information, was included in CNVIntegrate. Occurrences of CNA events are calculated for each gene and divided into groups based on site and histology of the respective tumor. As the number of tested samples for each cancer site might potentially differ and because such information was absent in the downloaded data from COSMIC, the population frequency of CNAs was manually curated in this study to be included in CNVIntegrate.

#### CCLE dataset

The CCLE is an ongoing collaboration between the Broad Institute and the Novartis Institutes, consisting of genomic and pharmacological data from 1457 human cancer cell lines, as of 2019 (35). The log_2_ CN values of 23 316 genes spanning 1043 cell lines were imported into CNVIntegrate. The samples for each cell line belonged to different cancer types, and therefore, the cell line annotation information from CCLE was used to classify the 1043 cell lines. For ease of interpretation, log_2_ CN values were then transformed into absolute CN values where a cell line was classified as a CN gain cell line only if its absolute CN was higher than 2.3. Similarly, it was considered as a CN loss cell line if its CN value was lower than 1.7. CNA frequency for each cancer was then calculated based on a predefined CNV gain/loss condition.

#### Cancer Gene Census

CGC (40) is an expert-curated guide built by exhaustive integration of all available literature and is commonly used across medical reports, pharmaceutical development and basic biological research as a standard annotation resource. CGC annotations, with information on classification of the genes, their role in cancer and molecular information depicting dominant or recessive status, were added to CNVIntegrate. With the purpose of CNV-gene mapping, a gene set consisting of 711 unique genes, complemented with CGC annotation, was created in this study, to be incorporated in the database.

### Gene-query function

For the query function (Figure 2), the database supports user queries by either gene symbols or human genome (GRCh37) chromosomal location. CNVIntegrate allows three types of user query formats (Supplementary Figure 1a). For batch queries, the database accepts a .txt or .csv file as input. For single gene queries, the autocomplete function allows the user to choose from options for the gene symbol that is keyed in. The gene search parameter is not case sensitive (e.g. ERBB2 or erbb2 are both valid entries for gene *ErbB2*), such that the system will automatically capitalize user input, to reduce query error. To obtain the CNV information of an entire chromosome, users are required to specify only the chromosome number as the query (e.g. chr1), while if the CNV information of a specified chromosomal region is of interest, the user can key in the chromosome number along with the start and ending position of the respective region (e.g. 1:10 123–50 123). Gene-based queries will allow the user to choose from a list of healthy populations to obtain a CNV frequency comparison analysis for the queried gene. Other available information includes the gene’s CGC annotation, its CNV distribution across different tissues, and the absolute CN in breast cancer cell lines in TWBC and different CCLE and COSMIC cell lines. A total of 35 055 gene transcripts are integrated into the database, among which 22 155 unique genes contain overlapping CNVs from healthy, cancer-free individuals. The gain and loss frequencies of 14 975 genes are available in TWCNV and ExAC, providing CNV information on ∼75 727 individuals without apparent illness. The TWCNV dataset alone hosts 26 129 unique genes, while 15 673 autosomal protein coding genes are available in the ExAC dataset.

### CNV profile function

To access the CNV profile of a specific cancer, users can select the cancer type (Supplementary Figure 1b) from the Cancer tab in the home page (Figure 2). It is designed as a four-level hierarchy of options from organs, organ sub-sites, primary histology and sub-histology. The primary site corresponds to the original site of the tumor. The user is allowed a further specification of the secondary site. CNVIntegrate consists of CNV profiles for 70 cancer types spanning 30 different tissues in humans, considering only primary sites and primary histology, while expanding to 419 cancer subtypes with the inclusion of secondary sites and secondary histology. Downloadable tables embedded with default sorting and filtering functions are also displayed on the home page so that users can easily retrieve and download required information in .csv format.

### Analysis function

The web site’s analysis function (Figure 2) allows systematic comparisons of CNV frequencies from different ethnic populations using statistical procedures. The analysis function aims to aid the user in identifying frequent CNV regions in a specific population through the implementation of a streamlined workflow. To utilize this function, users are first required to choose from a list of reference datasets (at most three datasets; two from healthy populations and one from cancer patients) to conduct the comparisons (Supplementary Figure 1c). Subsequently, the function also requires a user-prepared dataset with CNV information conforming to the CNVIntegrate format. Analysis results identify genes from the user’s list that have significant differences in CNV amplification/deletion frequencies between the dataset provided and the user-specified TWCNV and/or ExAC and/or specified cancer database. The *P*-values are generated by Fisher’s test (Supplementary Materials). Also, a Bonferroni-corrected *P*-value is reported.

## Results

### Database interface

CNVIntegrate offers a comprehensive database with compiled CNV/CNA data from both healthy people (TWCNV and ExAC) and cancer patients (TWBC, CCLE and COSMIC) from multiple populations. The database houses CNA profiles for 419 cancer types. It helps users, through two main functions, the query function and the analysis function, to identify key genes with significant roles in initiation, progression and prognosis of cancer, with respect to the part that CNV plays as a clinical biomarker. The web site communicates with the database via object-relational mapping provided by Django and utilizes SQL language for structured manipulation of genomic data.

### TWCNV: detection of CNVs in the Taiwanese population and comparison to other populations

**Figure 3.**
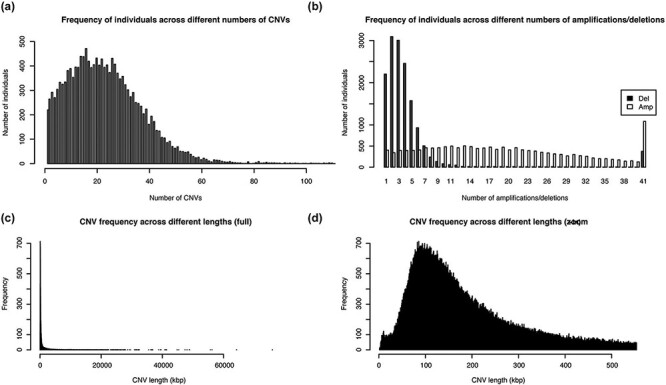
Distribution of the number and length of CNVs across the 15 829 Taiwanese people in TWCNV (a) Distribution of CNV counts in the population. (b) Distribution of duplication and deletion counts separately in the population. In (a) and (b), the number of individuals (y-axis) were plotted against the CNV counts (x-axis). (c) Distribution of lengths of the identified distinct CNVs. (d) Partial distribution of lengths of the identified distinct CNVs. In (d), CNV lengths ranging from 1 to 500 kb were further enlarged from the left figure. In (c) and (d), the unit of CNV lengths was kilobase pairs (kb) on the x-axis, and the exact CNV counts were shown on the y-axis.

The healthy cohort from Taiwan (TWCNV) consists of 476 247 CN alterations identified on autosomal chromosomes mapped to more than 20 000 genes from ∼16 000 volunteers with a mean age of 48.6 years (range 30–70). Table 2 lists the demographic statistics of the study population. The average mean and median CN segment counts per individual were found to be 30 and 22, respectively, with no detected CNVs in 99 samples (Figure 3a) and a maximum CNV count of 2709 CNVs detected in just one sample (not shown in the figure). As shown in Figure 3b, 65% of the total identified CNVs were deletions, while 34% were duplications. The sizes of the detected distinct CNVs (CNVs with different start or end positions) ranged from 1.8 kb to 75 Mb (mean size = 460.2 kb and median size = 172.1 kb; Figure 3c and d).

**Table 2.** Demographic characteristics of the healthy samples from the Taiwanese cohort

Variables	Number	Percentage (%)
Gender		
Female	8001	50.55
Male	7828	49.45
Total	15 829	100
Age		
30	386	2.44
31–40	4124	26.05
41–50	4285	27.07
51–60	4199	26.53
61–70	2835	17.91
Total	15 829	100
Residence		
North	4751	30.01
Central	5022	31.73
South	5891	37.22
East	159	1.00
Outlying Islands	6	0.04
Total	15 829	100
Ancestry		
Southern Fujian	11 091	70.07
Hakka	1538	9.72
Mixture of Southern Fujian and Hakka	831	5.25
Other regions of China	2312	14.61
Missing	57	0.36
Total	15 829	100

**Figure 4.**
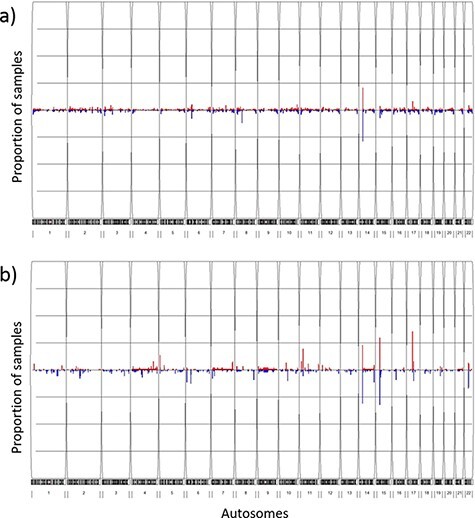
Frequency plots of CNVs in the TWCNV and HapMap datasets Red color represents duplication while blue color represents deletion. The x-axis contains the 22 autosomes, and the y-axis shows the proportion of samples showing CNVs in the two datasets. (a) Frequency plot of CNVs in 15 829 Taiwanese individuals. (b) Frequency plot of CNVs in 87 Asians in the HapMap dataset.

The overall CNV profile (Figure 4a) displayed less than 5% CNV frequency in most of the CNV segments, with the highest duplication (20.9%) and deletion (29.0%) frequencies at the 14q11.2 chromosome location. The genes that encompassed the CNVs at this location (*OR4Q3, OR4M1, OR4N2, OR4K3, OR4K2, OR4K5*, and *OR4K1*) belonged to the olfactory receptor family. Other CNV segments with relatively high frequency (>5%) included the 8p11.22 deletions, the 15q11.1-q11.2 deletions, the 6p21.32-p21.33 deletions and the 17q12 duplications. Genes enriched in these regions mostly belonged to pseudogenes with no known essential functions. We further compared CNV profiles from TWCNV with Hapmap. Repeated CNVs were found in the 14q11.2, 15q11.1-q11.2 and 17q12 chromosomal regions, with higher frequencies in both the TWCNV and the HapMap Asian samples (Figure 4b). The five regions with higher CNV rates were also discovered in the genomes of 155 Chinese control individuals on the SNP 6.0 array platform. In addition to Asian populations, the genomes of 490 Ashkenazi Jewish control individuals on the SNP 6.0 array platform were used for comparison. The same regions with high CNV frequencies were still visible.

### Example 1: query function applied to *ERBB2*

The *ERBB2* gene is mutated in 14.05% of breast carcinoma patients, with amplification present in 11.18% (48). Overwhelming evidence from numerous studies indicates that amplification or overexpression of *ERBB2* disrupts normal cell control mechanisms and gives rise to aggressive tumor cells (49). Patients with ERBB2-overexpressing breast cancer have substantially lower overall survival rates and shorter disease-free intervals than patients whose cancer does not overexpress ERBB2 (50). Moreover, overexpression of ERBB2 leads to increased breast cancer metastasis (51). Here, we use ERBB2 to demonstrate the query function of CNVIntegrate.

**Figure 5.**
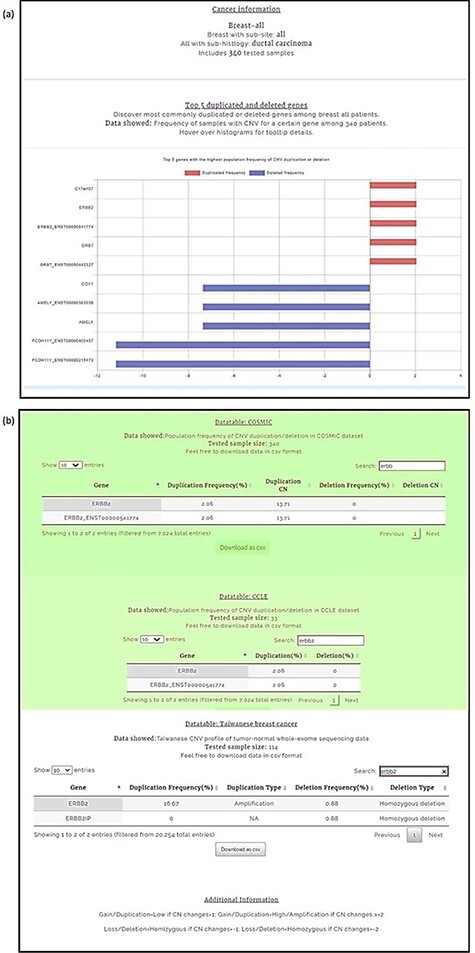
Breast carcinoma profile from the four-level hierarchy query system (a) Top five genes with the highest population frequency of CNV duplication or deletion. (b) Population frequency of CNV duplication/deletion in the CCLE, COSMIC and TWBC datasets.

**Figure 6.**
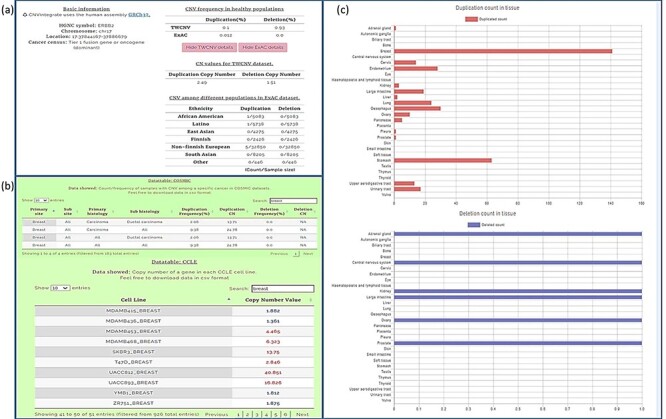
Query results for *ERBB2* gene showing CNV frequencies in both healthy populations and cancer populations (a) CNV frequency/counts of the *ERBB2* gene in the TWCNV (healthy) dataset and CNV frequency of *ERBB2* among different populations (healthy) in the ExAC dataset. (b) Count/frequency of breast cancer samples with CNV in the COSMIC dataset; CN of the *ERBB2* gene in CCLE breast cell lines. (c) Duplication and deletion counts of the *ERBB2* gene in different tissues.

We conducted our query in two steps. First, the CNA profile of breast cancer was selected from the four-level hierarchy (Supplementary Figure 1b), which listed *ERBB2* among the top five genes (Figure 5a) that exhibits duplication frequency of 2.06, 2.06 and 16.67% in COSMIC, CCLE and TWBC, respectively (Figure 5b). The gene symbol was then used as a query (Supplementary Figure 1a). The CNV frequencies (amplification and deletion) in TWCNV and ExAC were very low (Figure 6a), exhibiting neutrality in healthy cohorts. Furthermore, the tissue distribution charts showed that ERBB2 has the most CN duplication events in breast tissue in comparison to other tissues (Figure 6c); however, no deletion events were reported. This was further confirmed by both COSMIC and CCLE outputs identifying ERBB2 as possessing very high CN duplications among patients with breast cancer (Figure 6b).

These query results of CNVIntegrate conform to the findings from HER-2 breast cancer research over the years (52, 53), thereby establishing their reliability. Also, patients with *ERBB2*-negative tumors who have received adjuvant chemotherapy (cyclophosphamide, methotrexate, 5-fluorouracil, and prednisone [CMFP]) have shown a significantly greater rate of disease-free survival in response to therapy than patients with *ERBB2*-positive tumors, indicating that overexpression of ERBB2 plays a role in chemotherapy resistance (53). Hence, developing ERBB2-targeting strategies to improve the therapy of ERBB2-overexpressing breast cancer remains a high priority. During the last decade, several exciting techniques have been developed to target ERBB2. Although some are still under investigation, many studies have shown that these ERBB2-targeting techniques not only inhibit tumor growth, but also lead to chemo-sensitization of ERBB2-overexpressing cancer cells (53).

### Example 2: analysis function applied to lung adenocarcinoma

**Figure 7.**
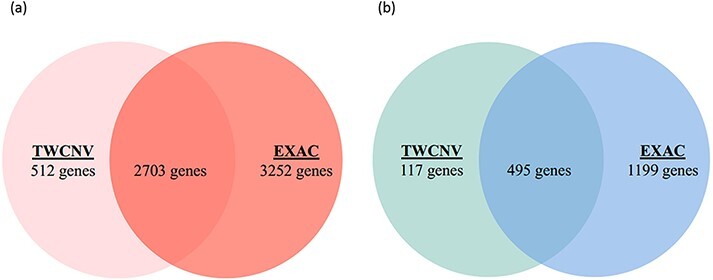
Venn diagrams of significant gene counts from the analysis function (a) Significant CN gains (Red) were observed in 2703 genes among lung adenocarcinoma patients from both TWCNV and ExAC populations. (b) Significant CN losses (Blue) were observed in 495 genes among lung adenocarcinoma patients from both TWCNV and ExAC populations.

Lung adenocarcinoma is the most common form of non-small cell lung cancer (NSCLC) and accounts for roughly 40% of lung neoplasms (54). Characterizing its genomic profile is crucial as NSCLC is strongly genetically disposed. Lung adenocarcinomas that are responsive to epidermal growth factor receptor (EGFR) tyrosine kinase inhibitors possess *EGFR* mutations and often increased EGFR CNs (55, 56). By comparison of the mutant and normal *EGFR* alleles, a preferential amplification of the mutant allele has been observed and EGFR overexpression was found to be strongly associated with this amplification (57). In conjunction with clinical traits and manifestations, CNVs have recently evolved as promising biomarkers that could assist in selecting clinical interventions (58). Supplementary Figure 1c shows a screen shot of the ‘Analysis function’ page with an input dataset (EGFR.csv) conforming to the required format. The input dataset consisted of a list of genes (∼28 000 genes) along with their gain and loss counts from 542 lung adenocarcinoma patients that was extracted from this study per se and has originated from the COSMIC dataset. The analysis function reports the list of genes with significant differences in CNV amplification/deletion frequencies between the dataset provided and the selected control population. By setting a Bonferroni correction threshold of 0.05 (*P*-value), 3215 and 612 genes were reported with CN gain (Figure 7a) and CN loss (Figure 7b), respectively, when compared with the TWCNV dataset. Likewise, for the ExAC dataset, 5955 and 1694 genes were reported with significant differences in CN gain and loss, respectively (Figure 7). The genetic abnormalities linked to risk of lung cancer would be better analyzed and understood if studied in the context of signaling pathways, rather than focusing on individual factors. Several pathways with major components have their functions altered in lung cancer, and these pathways are emerging as having considerable importance with regard to targeted therapy. Therefore, to characterize the biological functions implicated by the CNV-driven genes, Ingenuity Pathway Analysis (IPA) (59) was carried out to describe gene–gene interaction networks and canonical pathways. The genes with significant CN differences from both TWCNV and ExAC populations were isolated (2703 CN gains and 495 CN losses) and fed in IPA (https://www.qiagenbioinformatics.com/products/ingenuitypathway-analysis) to obtain a total of 53 canonical pathways that were deemed to be significantly enriched with CN genes (*P* < 0.001). A comprehensive overview of the distribution of CNV genes, from the significant signaling pathways using 542 lung adenocarcinoma patients, reported genes that have already been verified as key biomarkers in lung adenocarcinoma progression such as *EGFR, Kras* and *MET,* which had CN gain frequencies between 22 and 25% (results not shown). The five pathways with the most significant *P*-values included small cell lung cancer signaling, molecular mechanisms of cancer, synaptogenesis signaling pathway, axonal guidance signaling pathway and G-protein coupled receptor signaling pathway (Table 3). As reported by IPA, functions such as cell death and survival, cellular development and cellular growth and proliferation are associated with both small cell lung cancer signaling pathway and molecular mechanisms of cancer pathways. Synaptogenesis signaling pathway has been reported to be s activated by synaptic growth factors that are promoted by Protein kinase Cϵ (PKCϵ) (60), where PKCϵ has been observed to play a role in the JNK activation in human lung cancer cells (61). Several semaphorin genes in the axonal guidance signaling pathway (e.g. *SEMA5A, SEMA6A*) has also been identified in previous studies as potential therapeutic targets for NSCLC patients (62). Finally, signaling pathways controlled by G-protein coupled receptors promote proliferation, survival, cell migration, angiogenesis, inflammation and subversion of the immune system and their overexpression potentially contribute to the malignant transformation of cells (63). Furthermore, one major common function implicated by the top significant networks is cell survival regulation via AKT signaling, which has been extensively studied and targeted in lung cancer therapy (29).

**Table 3.** Top 10 significant pathways of 2703 CN gain genes and 495 CN loss genes of lung adenocarcinoma patients

Pathway	-log *P*-value	Genes count	Percentage (%)
Small cell lung cancer signaling	8.63	31	40
Molecular mechanisms of cancer	7.94	92	23
Synaptogenesis signaling pathway	7.24	76	23
Axonal guidance signaling	6.59	103	23
G-protein coupled receptor signaling	6.25	66	23
Senescence pathway	5.6	64	22
Colorectal cancer metastasis signaling	4.91	58	22
Regulation of the epithelial-mesenchymal transition pathway	4.42	45	23
G beta gamma signaling	4.35	34	25
IL-8 signaling	4.06	46	22

## Discussion

CNVIntegrate is a comprehensive CNV and CNA resource for both healthy and cancer-affected individuals, respectively, with data gathered from public databases from different ethnic populations. While a majority of CNVs/CNAs are benign, prior studies have verified that they can sometimes drive the progression of heritable and somatic human diseases such as cancer, neuropsychiatric disorders and viral infection. One of the barriers to using the massive amount of accumulated cancer genomic information is how to prioritize and interpret the data on CNVs/CNAs. The effort of linking genetic observations with relevant clinical information is further complicated by the heterogeneous and unstructured forms of the available data. For instance, the CNA data provided by CCLE are either in CEL format (probe intensity calculations) from Affymetrix DNA microarray analyses or in unformatted text format comprising roughly 23 000 pieces of unstructured information. For users without bioinformatics proficiency or lacking experience with sophisticated computational methodologies, additional technical support is required for data processing before it can be applied in genetic studies. Moreover, obtaining computational assistance is often not financially feasible in small to moderate-sized research groups, creating a methodological barrier in CNV studies (64). These are a few of the many reasons why valuable CNV resources fail to be utilized to their full potential. CNVIntegrate was developed in an attempt to resolve such issues. It allows the storage, retrieval and analysis of genomic data in a meaningful and structured manner that assists biomedical researchers in performing efficient data interpretation. Moreover, it provides statistically comparable metrics to predict the functional impact of a CNV/CNA, thereby assisting users in prioritizing significant causal CNVs for simplified data mining and data retrieval for future CNV research.

The two healthy population CNV datasets that were integrated into CNVIntegrate utilize classic, yet different approaches toward CNV identification. TWCNV was constructed from SNP arrays, whereas ExAC utilized harmonized WES data. Array-based approaches are cost-effective and reliable methods of large-scale analysis, while WES offers the ability to detect smaller variations with more accurate break-point identification due to higher coverage and resolution (65). Despite the rapid advancements pertaining to various CNV calling algorithms, previous studies raised cross-platform consistency as a concern, debating the necessity of platform-specific normalization. Ruderfer *et al.* conducted a comparison using a subset of 10 091 individuals from ExAC that had both WES data and high-quality CNV calls from genotyping arrays (66) and reported that although more CNVs were discovered by WES, 78% of longer CNV regions (defined as regions intersecting with more than 20 target genes) that were detected by arrays overlapped with exome sequenced CNVs. On average, 83% of the exons were included in calls from both technologies (67), indicating reproducible results by both platforms when fortified by appropriate QC measures. Such inferences about the consistency of cross-platform results were supported by other studies as well (68). In this study we have utilized more efficient filtering and more comprehensive assessments of CNVs in comparison to these prior studies, and therefore, CNVs called by TWCNV and ExAC do not require further normalization to avoid the possible introduction of false negatives.

The importance of TWCNV stems from the fact that the occurrence of CNV frequencies, or lack thereof, among generally healthy people constitutes an important evidence base when the clinical impact of such variations is considered (69). The classification of a variant often relies on its prevalence in presumably healthy unaffected individuals (70), so as a large database, with gene-level frequencies from nearly 75 000 individuals, the data-driven information from TWCNV is critical for excluding common variants that are less likely to be deleterious or pathogenic. Also, when CNV calling results from TWB arrays were compared to that of three external datasets (HapMap Asian samples, Chinese control samples on the SNP 6.0 array platform and 490 Ashkenazi Jewish control samples (Non-Asian) on the SNP 6.0 array platform), the common CNVs of the 14q11.2, 8p11.22, 15q11.1-q11.2, 6p21.32-p21.33 and 17q12 chromosomal regions were observed in all the datasets.

## Conclusion

The CNVIntegrate database is the only web-based system that hosts CNV and CNA data from both healthy populations and cancer patients and permits input of user data when performing statistical comparisons between CNV frequencies of multiple populations. The major target audience and expected users of this database are mainly cancer biology researchers that rely on an interactive graphical user interface. Through this effort we have eliminated the need for bioinformatics proficiency and the technical support that would typically be required for accessing integrated CNV information. In summary, CNVIntegrate provides a ready-made dataset that is structured and interpretable and has an easy query function, an equally easy download system and an online submission analysis function.

## Supplementary Material

baab044_Supp
