# Supplementary material for: CNVIntegrate: the first multi-ethnic database for identifying copy number variations associated with cancer
Source: Database (Oxford). 2021 Jul 14;2021:baab044. doi: 10.1093/database/baab044 (PMC8278790; doi:10.1093/database/baab044)
Supplement: baab044_Supp [file baab044_supp.zip › Supplementary figure 1.docx]

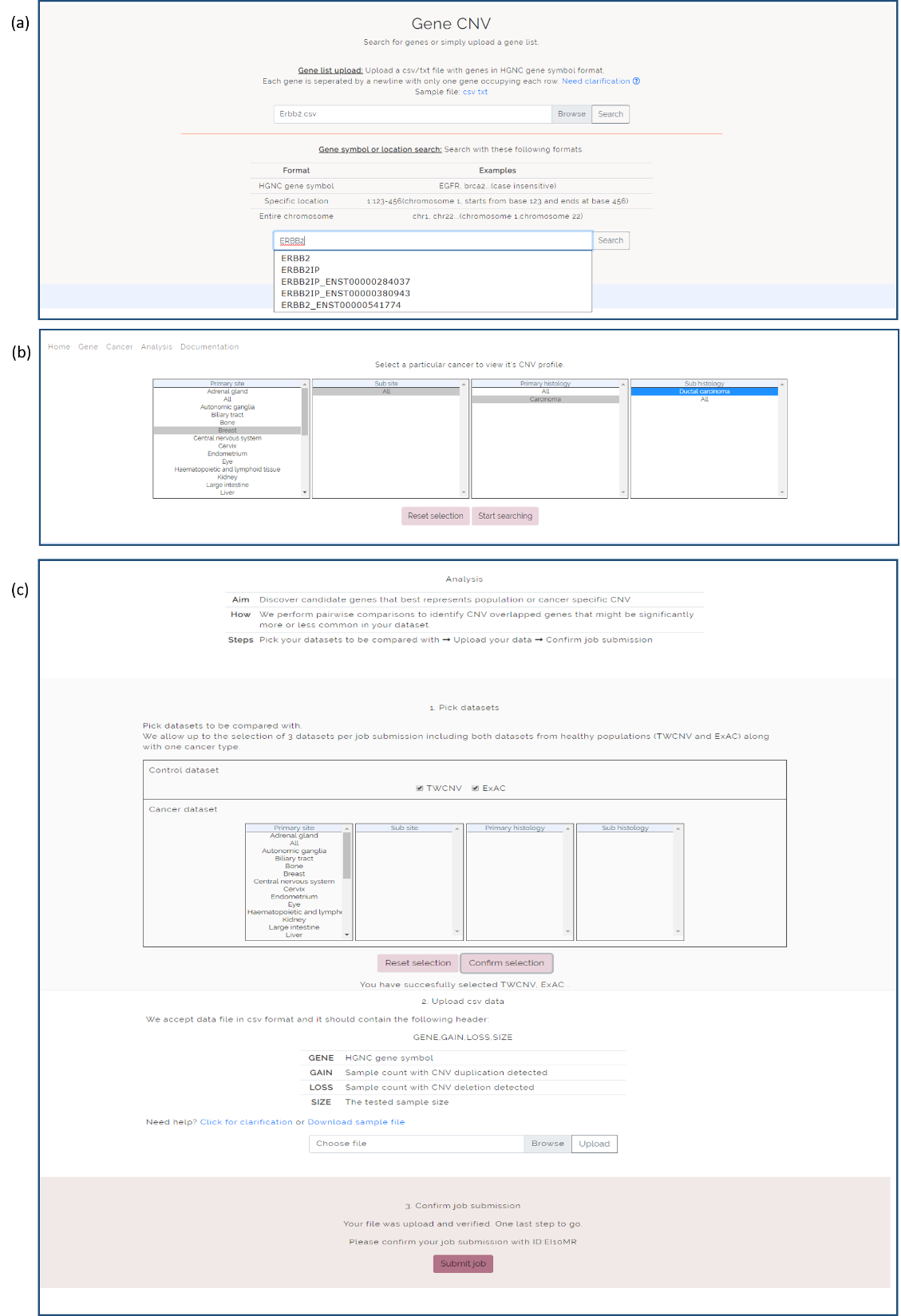


Supplementary figure 1. **Screen Shots for functions of CNVIntegrate.** (a) Screen Shot of Gene Query function. (b) Screen Shot of Cancer Profile function. (c) Screen Shot of Analysis function
