# Supplementary material for: CNVIntegrate: the first multi-ethnic database for identifying copy number variations associated with cancer
Source: Database (Oxford). 2021 Jul 14;2021:baab044. doi: 10.1093/database/baab044 (PMC8278790; doi:10.1093/database/baab044)
Supplement: baab044_Supp [file baab044_supp.zip › Supplementary Materials.docx]

**Supplementary Materials**

**Analysis Function**

The website’s back-end view logic is responsible for performing all statistical computation and thus the generation of output result files. For each pairwise comparisons of gene CNV frequency, two 2 x 2 contingency tables was created as follow, so that Fisher’s exact test could be performed with CN gain frequency and CN loss frequency tested separately.

|  | Sample with CN gain | Sample without CN gain |
| --- | --- | --- |
| User tested sample size (X) | k | X-k |
| Database tested sample size (Y) | m | Y-m |
|  |  |  |
|  | Sample with CN loss | Sample without CN loss |
| User tested sample size (X) | l | X-l |
| Database tested sample size (Y) | n | Y-n |

The p-value generated determine the significance level of the differences observed in CNV frequency among the two distinct populations. It was followed by a Bonferroni correction to reduce the chances of obtaining false-positive results and corrected the p-value was also recorded. A gene pair-wise comparison with p-value<0.05 before Bonferroni correction is considered significant thus will be selected to be included in the output results.
